# Recombinant Expression and Characterization of a Novel Thermo-Alkaline Lipase with Increased Solvent Stability from the Antarctic Thermophilic Bacterium *Geobacillus* sp. ID17

**DOI:** 10.3390/ijms25147928

**Published:** 2024-07-19

**Authors:** Diego Salas-Bruggink, Hardy Guzmán, Giannina Espina, Jenny M. Blamey

**Affiliations:** 1 Fundación Biociencia, José Domingo Cañas 2280, Santiago 7750132, Chile; dsalas@bioscience.cl; 2 Facultad de Química y Biología, Universidad de Santiago de Chile, Alameda 3363, Santiago 9170022, Chile; hardy.guzman@usach.cl

**Keywords:** esterase, protein purification, biocatalyst, biotechnology, extremophile, extremozyme

## Abstract

Lipases are enzymes that hydrolyze long-chain carboxylic esters, and in the presence of organic solvents, they catalyze organic synthesis reactions. However, the use of solvents in these processes often results in enzyme denaturation, leading to a reduction in enzymatic activity. Consequently, there is significant interest in identifying new lipases that are resistant to denaturing conditions, with extremozymes emerging as promising candidates for this purpose. Lip7, a lipase from *Geobacillus* sp. ID17, a thermophilic microorganism isolated from Deception Island, Antarctica, was recombinantly expressed in *E. coli* C41 (DE3) in functional soluble form. Its purification was achieved with 96% purity and 23% yield. Enzymatic characterization revealed Lip7 to be a thermo-alkaline enzyme, reaching a maximum rate of 3350 U mg^−1^ at 50 °C and pH 11.0, using p-nitrophenyl laurate substrate. Notably, its kinetics displayed a sigmoidal behavior, with a higher kinetic efficiency (*k*_cat_/*K*_m_) for substrates of 12-carbon atom chain. In terms of thermal stability, Lip7 demonstrates stability up to 60 °C at pH 8.0 and up to 50 °C at pH 11.0. Remarkably, it showed high stability in the presence of organic solvents, and under certain conditions even exhibited enzymatic activation, reaching up to 2.5-fold and 1.35-fold after incubation in 50% *v*/*v* ethanol and 70% *v*/*v* isopropanol, respectively. Lip7 represents one of the first lipases from the bacterial subfamily I.5 and genus *Geobacillus* with activity and stability at pH 11.0. Its compatibility with organic solvents makes it a compelling candidate for future research in biocatalysis and various biotechnological applications.

## 1. Introduction

Triacylglycerol lipases (E.C 3.1.1.3), commonly referred as lipases, are a family of serine hydrolases that belong to the group of lipolytic enzymes that catalyze the ester carboxyl bond of triglycerides [1]. Lipases are distinguished from other carboxylesterases by their preference for hydrolyzed long-chain substrates that are usually insoluble in water and their enzymatic activation in the presence of lipid–water interfaces, known as interfacial activation [2,3].

These enzymes have diverse applications in industry, being currently used in drug synthesis, food processing, biofuel production, pulp refinement, detergent formulation, and bioremediation [2,4,5]. These applications are related to the ability of lipases to biocatalyze hydrolysis as well as synthesis reactions in organic solvents. Examples of such reactions include esterification and transesterification in the presence of high concentrations of alcohols [6,7] or even more complex reactions, such as Michael reactions, aldol condensations, and epoxidations, among others [8,9,10,11].

However, most studies associated with lipase catalysis have reported low yields due to enzyme denaturation under the reaction conditions [8,12]. For instance, in the application of lipases for biofuel production, high concentrations of methanol or ethanol are required, but these concentrations lead to rapid unfolding and inactivation of the enzyme within a few cycles of the process [13,14,15]. Similarly, in drug synthesis involving lipases, solvents such as ethanol, isopropanol, dimethyl sulfoxide (DMSO), acetonitrile, acetone, and hexane are used, with concentrations ranging from 20% (*v*/*v*) solvent–water to completely anhydrous conditions. There are also cases where reactions must be performed at high temperatures, typically between 40 °C and 60 °C [6,7,8,9,10,11,16]. In these applications, the challenge is maintaining the functional structure and stability of the enzyme and preventing its inactivation during the synthesis of the compounds of interest.

Consequently, the purification of lipases from thermophilic microorganisms is of significant interest, as their enzymes have already adapted to maintain activity at elevated temperatures. Furthermore, thermophilic enzymes have demonstrated not only thermostability but also resilience in chaotropic agents, organic solvents, and high salinity [17,18].

Deception Island is the caldera of an active volcano located in the southwestern part of Bransfield Strait, between the Antarctic Peninsula and the South Shetland archipelago [19]. This island contains geothermal sites such as Fumarole Bay, which exhibits emission temperatures of up to 110 °C. From environmental samples collected at this site, it was possible to isolate a thermophilic bacterium belonging to the *Geobacillus* genus, identified as *Geobacillus* sp. ID17 [20]. This microorganism grows at 50–70 °C and possesses different thermophilic enzymes, including thermostable lipases [20,21]. However, production and purification from the native microorganism resulted in very low yield, preventing further biochemical characterization [21]. Due to these limitations, the genomic DNA from *Geobacillus* sp. ID17 was sequenced, and several genes encoding for lipases/carboxylesterases were identified through comprehensive bioinformatic analysis. One interesting gene, identified as *lip7*, was found to belong to subfamily I.5 of bacterial lipases, which is characterized by high temperature stability and resistance to organic solvent denaturation [13,14,22,23,24,25,26].

In this study, the obtaining of the recombinant version of a novel thermophilic lipase in functional soluble form is described, along with its purification and biochemical characterization. To the best of our knowledge, Lip7 represents one of the first thermo-alkaline lipases from bacterial subfamily I.5 and genus *Geobacillus*, exhibiting activity and stability at pH 11.0. Kinetic characterization allows determination of the enzyme preference for medium-chain substrates and the identification of its interfacial activation. Moreover, Lip7 showed high stability in the presence of organic solvents and even exhibits enzymatic activation up to 2.5-fold in 50% *v*/*v* ethanol and 1.35-fold in 70% *v*/*v* isopropanol. The results of this work highlight the biotechnological potential of the novel thermophilic lipase Lip7, suggesting that this extremozyme could be a promising candidate for further research and biotechnological and industrial applications.

## 2. Results

### 2.1. Overexpression and Purification of the Recombinant Lipase

The lipase-encoding gene (*lip7*) from *Geobacillus* sp. ID17 was successfully expressed in a soluble and catalytically active form in *E. coli* C41 (DE3). This strain was selected after initial trials using *E. coli* BL21 (DE3) resulted in very low soluble expression and reduced enzyme activity (Figure S1).

Lipases from subfamily I.5 of microbial lipolytic enzymes have been reported to possess calcium and zinc binding sites associated with high-temperature stability [22,23,24,25,26]. Therefore, for the first purification step of Lip7, the soluble crude extract was heated at 60 °C for 10, 30, 60, and 120 min after preincubation with CaCl_2_, ZnCl_2_, or a combination of both salts (Figure S2). As a result, preincubation in CaCl_2_ significantly increased the thermostability of Lip7, whereas preincubation in ZnCl_2_ did not show an improvement in Lip7 stability, and a combination of both salts showed a similar effect to incubation with CaCl_2_. This suggests that the addition of calcium is sufficient to enhance the thermal stability of Lip7, and that preincubation of the crude extract with 0.5 mM CaCl_2_ for 15 min at 25 °C, allowed for an efficient heat-denaturation treatment at 60 °C for 30 min as an initial purification step.

**Figure 1:**
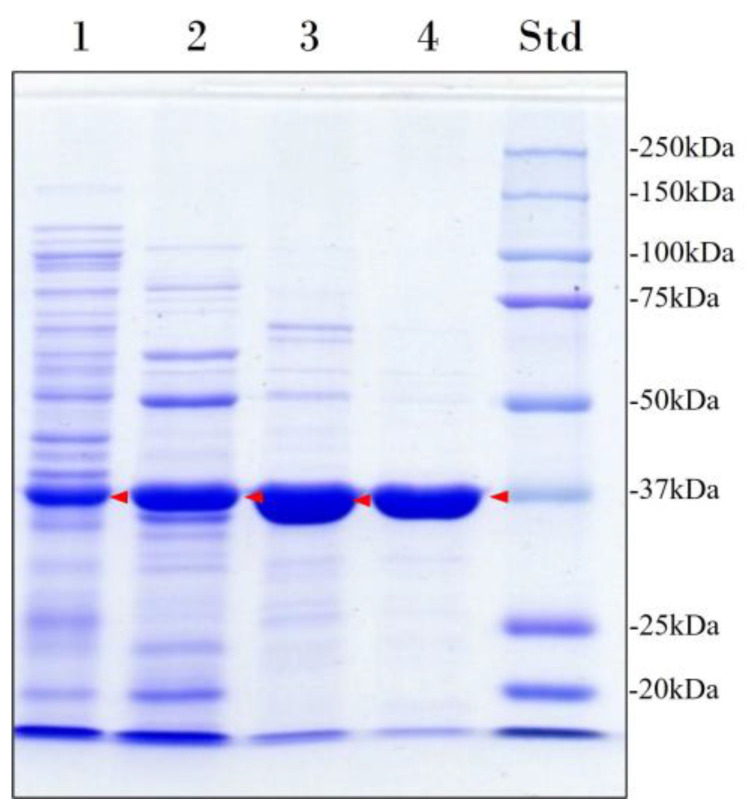
Electrophoretic analysis of the heterologous overexpression and purification of Lip7. Lane 1: Cell-free soluble crude extract; Lane 2: heat-denaturation treatment at 60 °C for 30 min after preincubation in CaCl_2_; Lane 3: pooled fractions with lipolytic activity eluted from the cation exchange chromatography; Lane 4: pooled fractions with lipolytic activity eluted from the size-exclusion chromatography. Lane Std: molecular weight marker (Bio-Rad Precision Plus Protein™ Kaleidoscope™ pre-stained protein standard). The protein concentration loaded in each well is 15 μg and the band corresponding to Lip7 is highlighted by red arrows. Purity percentages of each purification step were 40%, 52%, 83%, and 96%, respectively, as determined with ImageJ software version 1.54i [28].

The recombinant enzyme was purified to homogeneity (96% purity) by heat treatment followed by cation exchange chromatography and size-exclusion chromatography (Figure 1, Table 1). In addition, size-exclusion chromatography was performed to assess the molecular mass and oligomeric state of the enzyme. The result obtained was 47 kDa, indicating that the enzyme is a monomer. However, this result differs from the apparent molecular mass of 37 kDa determined by sodium dodecyl sulfate–polyacrylamide gel electrophoresis (SDS-PAGE) (Figure 1) and the theoretical molecular mass of 43 kDa predicted by Expasy ProtParam tool [27] from the translated *lip7* gene sequence without its signal peptide.

### 2.2. Characterization of the Recombinant Lip7

#### 2.2.1. Effect of pH and Temperature

**Figure 2:**
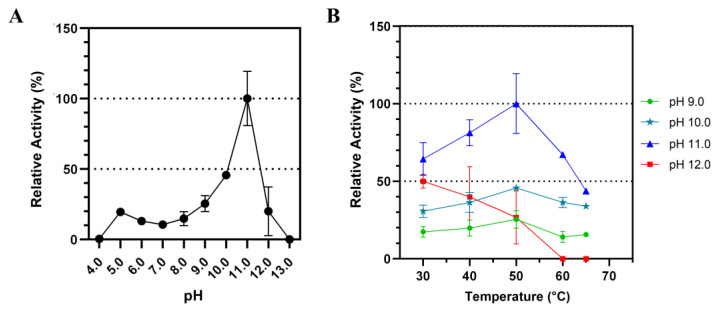
Influence of pH and temperature on the catalytic activity of Lip7 using Britton–Robinson universal buffer containing 2% (*v*/*v*) Triton X-100. (**A**) Optimum pH for purified Lip7 was assessed over a pH range of 4.0–13.0 at 50 °C. (**B**) Optimum temperature for Lip7 was evaluated in the range of 30–65 °C at various pH: 9.0 (green circle), 10.0 (teal star), 11.0 (blue triangle), 12.0 (red square). For each assay, 0.25 nM (0.011 μg mL^−1^) of purified Lip7 and 3.0 mM of pNPL substrate were used. Specific activity of the enzyme showing 100% relative activity was 3350 U mg^−1^. Error bars represent the standard deviation of the mean from three independent experiments.

The influence of different pH on Lip7 catalytic activity was examined over a pH range of 4.0–13.0 using p-nitrophenyl laurate (pNPL) substrate at 50 °C (Figure 2). The activity increased at higher pH until pH 11.0 was reached, after which it abruptly decreased (Figure 2A). Similarly, the activity increased with temperature until reaching 50 °C, but decreased thereafter (Figure 2B). This effect of temperature was consistent at pH 9.0, 10.0, and 11.0, but not at pH 12.0, where activity decreased with increasing temperature, indicating reduced thermostability at this pH. These results suggest that Lip7 is a thermo-alkaline lipase with maximum activity of 3350 U mg^−1^ at 50 °C, pH 11.0 with pNPL.

Thermal stability of Lip7 was evaluated at 40 °C, 50 °C, 60 °C, and 70 °C at pH 8.0 and pH 11.0, as the recombinant lipase is stored in buffer containing 50 mM Tris-HCl, pH 8.0, and its highest specific activity was obtained with Britton–Robinson universal buffer at pH 11.0.

**Figure 3:**
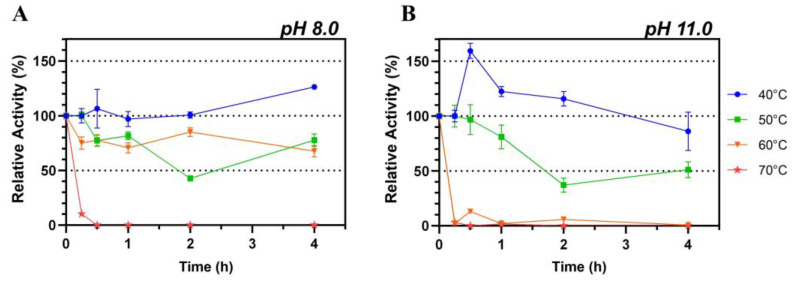
Thermostability of the purified Lip7. The enzyme was incubated at 40 °C (blue circle), 50 °C (green square), 60 °C (orange inverted triangle) and 70 °C (red star) at pH 8.0 (**A**) and pH 11.0 (**B**) for increasing lengths of time. Residual activity was measured under optimal conditions, at 50 °C, pH 11.0, using Britton–Robinson universal buffer containing 2% (*v*/*v*) Triton X-100. For each assay, 0.25 nM (0.011 μg mL^−1^) of purified Lip7 and 3.0 mM of pNPL substrate were used. Specific activity of the non-heated enzyme control showing 100% relative activity was 3350 U mg^−1^. Error bars represent the standard deviation of the mean from three independent experiments.

Lip7 exhibited high thermostability at 40 °C, 50 °C, and 60 °C at pH 8.0, maintaining more than 50% of its relative activity after 4 h of incubation. However, at 70 °C, the activity decreased sharply after only 15 min of incubation, leading to complete loss of activity (Figure 3A). Notably, when Lip7 was incubated at 40 °C, there was no loss of enzyme activity, and it even increased over 120% after 4 h of incubation.

Conversely, when thermostability was assessed at pH 11.0, the activity was only maintained at 40 °C and 50 °C, with an abrupt loss of activity at 60 °C and 70 °C within 15 min of incubation (Figure 3B). Similarly to observations at pH 8.0, incubation at 40 °C resulted in a relative increase in activity of more than 150% after 30 min, which gradually decreased to 75% after 4 h.

These results suggest that Lip7 is stable at elevated temperatures, but its thermal stability is influenced by the pH of the buffer solution used. Lip7 is thermostable up to 60 °C at pH 8.0 and up to 50 °C at pH 11.0. Additionally, it seems that enzymatic activation occurs at 40 °C, especially at pH 11.0, but also at pH 8.0.

#### 2.2.2. Stability against Organic Solvents

**Figure 4:**
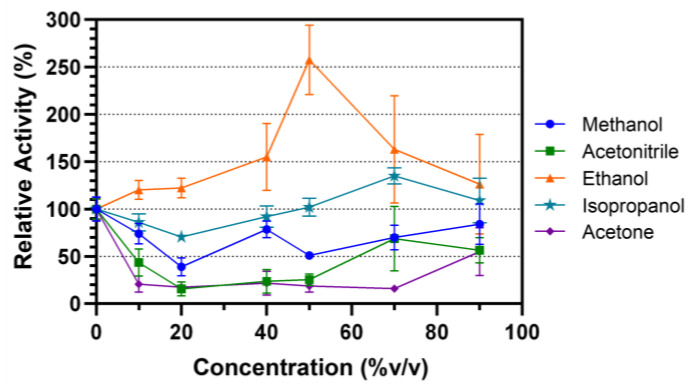
Organic solvent stability of the purified Lip7. The enzyme was incubated in different solvent–water concentrations (% *v*/*v*): 0%, 10%, 20%, 40%, 50%, 70%, and 90% of methanol (blue circle), ethanol (orange triangle), isopropanol (teal star), acetonitrile (green square), and acetone (purple diamond), for 30 min at 25 °C. Residual activity was measured under optimal conditions, 50 °C, pH 11.0 using Britton–Robinson universal buffer containing 2% (*v*/*v*) Triton X-100. For each assay, 0.25 nM (0.011 μg mL^−1^) of purified Lip7 and 3.0 mM of pNPL substrate were used. Specific activity of the non-incubated enzyme showing 100% relative activity was 3350 U mg^−1^. Error bars represent the standard deviation of the mean from three independent experiments.

Organic solvent stability of Lip7 was evaluated by incubating the enzyme in different solvent–water concentrations (% *v*/*v*) for 30 min at 25 °C (Figure 4). The selected solvents were miscible in water and ranged by polarity from higher to lower in the order of methanol, ethanol, isopropanol, acetonitrile and acetone. In methanol, the activity of the enzyme decreased relative to the non-incubated control, but maintained activity above 50% in most solvent–water concentrations. Acetone and acetonitrile decreased the relative activity of the enzyme to 30% after incubation in almost all solvent–water concentrations. However, in both cases, at 90% *v*/*v*, their activity increased to approximately 50%. Finally, Lip7 not only maintained its enzymatic activity when incubated in ethanol and isopropanol, but even underwent enzymatic activation. Lipolytic activity increased to a maximum of 250% at 50% *v*/*v* ethanol, and 135% at 70% *v*/*v* isopropanol. These results indicate that Lip7 is stable in organic solvents, as it maintained at least 30% of its activity under all conditions tested.

#### 2.2.3. Substrate Specificity

To further characterize the catalytic properties of the recombinant enzyme, the kinetic parameters of the hydrolysis of p-nitrophenyl acetate (pNPA, C2), p-nitrophenyl octanoate (pNPO, C8), p-nitrophenyl decanoate (pNPD, C10), pNPL (C12), p-nitrophenyl myristate (pNPM, C14), and p-nitrophenyl palmitate (pNPP, C16) were estimated at 25 °C and pH 8.0.

The saturation curve for each substrate was fitted to Michaelis–Menten and allosteric sigmoidal models (Table S1, Figure S3, Table 2). The allosteric sigmoidal model had a better fit and was consistent with the interfacial activation, corresponding to increased lipase activity at aqueous–lipid interfaces [3]. It is important to note that due to the increase in turbidity at high substrate concentration for long-chain substrates, it was not possible to saturate Lip7 with pNPP without interfering with the assay measurement. Nevertheless, with these results, it is possible to propose that Lip7 exhibits a preference for medium-chain substrates, especially pNPL, which exhibits the highest kinetic specificity (*k*_cat_/*K*_1/2_). Significant activity was also observed with pNPD, albeit with higher *K*_1/2_ (Table 2). However, the use of the allosteric sigmoidal model required careful data interpretation, which is further discussed in the next section.

## 3. Discussion

Lipases have diverse biotechnological and industrial applications, and consequently it is important to identify and study new enzymes that are well suited for these purposes [2].

Following the functional approach, which is based on the screening of enzymatic activities of interest from culturable microorganisms [29,30], it was possible to obtain several enzymes with lipolytic activity from the thermophilic bacterium *Geobacillus* sp. ID17, isolated from an environmental sample collected from Deception Island, Antarctica [20,21]. However, production and purification of the native enzymes from the thermophilic microorganism resulted in very low yield, preventing further biochemical characterization [21]. Due to these limitations, the genomic DNA from *Geobacillus* sp. ID17 was sequenced, and an interesting lipase encoding gene (*lip7*) was identified through comprehensive bioinformatic analysis and synthetized for recombinant overexpression in *E. coli.* It has been reported that lipases face a significant limitation in their heterologous expression, as they tend to form protein aggregates [31,32,33] that can be toxic to the host organism, triggering stress responses such as increased protease expression, inclusion body formation, loss of the expression vector, or cell death, thereby reducing production efficiency [34].

Therefore, in order to enhance soluble protein expression, the heterologous host *E. coli* C41 (DE3) was selected, as it is a BL21 (DE3) mutant characterized by its high tolerance to toxic protein overexpression [35]. This increased tolerance is attributed to a mutation in the *lac*UV5 promoter, which governs the expression of T7 RNA polymerase in strains derived from BL21 (DE3) [36,37]. This mutation weakens the *lac*UV5 promoter, thus reducing protein production levels, mitigating the toxic effects of recombinant overexpression. Although the use of C41 (DE3) strain has been reported in some publications, its application for heterologous expression of lipases is not yet widespread [38,39].

The purification of Lip7 was achieved by heat-denaturation treatment, followed by cation exchange and size-exclusion chromatography, which resulted in 96% purity and a 23% yield, with specific activity of 3350 ± 81 U mg^−1^ at 50 °C, pH 11.0 using pNPL substrate. A crucial step in this purification was the initial heat-denaturation treatment at 60 °C for 30 min after preincubation of Lip7 with 0.5 mM of CaCl_2_ for 15 min at 25 °C, which led to a 2.6-fold purification increase. This purification step presents several advantages in downstream processes, as it precipitates thermosensitive native proteins from *E. coli* while preserving the thermostable enzyme of interest [18,40,41]. It has been previously reported that lipases belonging to subfamily I.5 of microbial lipolytic enzymes, such as Lip7, possesses calcium and zinc binding sites that contribute to their high-temperature stability [22,23,24,25,26]. For instance, the thermostable lipase from *Geobacillus stearothermophilus* L1 exhibits an increase in stability of about 8–10 °C in the presence of calcium [23]. On the other hand, the role of zinc in the thermostability of lipases from subfamily I.5 varies, in some cases, its presence enhances stability, while in others, it may destabilize lipase activity [22,24,25]. Lip7 does not demonstrate an increase in thermostability in the presence of zinc (Figure S2).

It is worth noting that the yield of the second purification step (cation exchange chromatography), decreased from 50% to 21%. This is because Lip7 eluted from the column between 0.04 to 0.14 M NaCl; however, in order to ensure the highest purity of the recombinant enzyme, only the fractions with highest activity and no co-elution of other proteins, eluted between 0.04 and 0.08 M NaCl, which were selected for subsequent purification steps, despite compromising a better yield. For future optimization of the purification, the yield of the cation exchange chromatography step could be improved by using a shallow linear gradient or by evaluating a stepwise elution protocol at determined salt concentrations. The third step of purification (size-exclusion chromatography) also allowed to assess the molecular mass and oligomeric state of the enzyme. The result obtained was 47 kDa, while the molecular mass of the purified Lip7 determined by SDS-PAGE gave an apparent molecular mass of 37 kDa, revealing a discrepancy between the two methods. The differences could be attributed to certain properties of lipases, regarding SDS-PAGE, anomalous migrations have been described for hydrophobic proteins, especially membrane proteins, which have a higher affinity for detergents, and bind to SDS at a different rate that is often higher compared to the normal binding of standard proteins [42]. Since lipases are enzymes that are absorbed at water–lipid interfaces, they may exhibit similar behavior. This anomalous migration has been reported in other lipases, with Lip7 being no exception [22,43]. On the other hand, the differences observed in size-exclusion chromatography could be due to differences in the hydrodynamic radius between the protein of interest and the standards used [44]. Variations in the molecular mass obtained for lipases by size-exclusion chromatography have also been previously reported [32,33,43]. Despite these variations in molecular mass, the results obtained indicate that Lip7 is a monomeric enzyme. Monomeric lipases have been reported for other members of bacterial subfamily I.5, such as *Geobacillus* sp. T1 lipase [22].

According to the results obtained, Lip7 is a thermo-alkaline enzyme with maximum activity observed at 50 °C, pH 11.0. It exhibits high thermostability at 40–60 °C at pH 8.0 and 40–50 °C at pH 11.0, while lipases from the subfamily I.5 are known for their high activity at 50–70 °C and pH 8.0–10.0. To date, none of the lipases belonging to subfamily I.5 have been reported to possess activity at pH 11.0 [22,43,45,46,47]. Regarding stability, only *Geobacillus thermocatenulatus* BTL-2 lipase maintains approximately 85% of its activity after 12 h of incubation at 30 °C, pH 11.0, but its stability at higher temperatures has not yet been described [43]. On the other hand, lipases from other subfamilies within the genus *Geobacillus* do not exhibit activity at pH 11.0, but thermal stability of some enzymes has been reported. For instance, *Geobacillus thermodenitrificans* AV-5 lipase maintains near 100% activity after 30 min of incubation at pH 10.0 and 65 °C, while *Geobacillus stearothermophilus* FMR12 lipase exhibits 85% activity after 1 h incubation at pH 11.0 and 25 °C [48,49]. Therefore, to the best of our knowledge, Lip7 could be the first thermo-alkaline lipase from the subfamily I.5 and genus *Geobacillus* with activity and thermostability at pH 11.0. However, it is not the first thermo-alkaline lipase to exhibit these properties: the lipase from *Enterococcus faecium* MTCC5695 exhibits maximum activity at pH 10.8 °C and 40 °C, while *Staphylococcus* sp. ESW lipase shows maximum activity at pH 12.0 and 60 °C [50,51]. Additionally, lipase from *Staphylococcus* sp. ESW maintains 100% of its activity after 24 h incubation at room temperature at pH 11.0 and 12.0.

Thermo-alkaline lipases are needed in various industries, such as detergent manufacturing, paper production, leather processing, and bioremediation [4].

Regarding the saturation curves of Lip7 for substrates of different chain-lengths, a better fit to the allosteric sigmoidal model was observed, indicating behavior resembling typical allosteric effects. This is not commonly reported in recent determination of kinetic parameters for other lipases, where the data are directly fitted to the Michaelis–Menten model [13,52,53,54,55]. However, this phenomenon of increased activity, known as interfacial activation, has been extensively described in the last 50 years [3,56,57,58,59]. Lipases are esterases capable of hydrolyzing substrates under insoluble conditions, such as in emulsions or micelles, where they are absorbed at their interface. It is at these interfaces where the interfacial activation occurs, which refers to the significant difference in activity observed at the lipid–water interface compared to that observed with soluble free substrate [3]. Therefore, the reaction rate will not only depend on the substrate concentration but also on the physicochemical characteristics of the interface, where experimental variables affecting it, such as agitation, ionic strength, surface area, substrate surface concentration, temperature, and the use of detergents or surfactants, can significantly affect kinetic parameters and saturation curves [3,56,57,58,59].

In the experiments performed with Lip7, the use of 2% (*v*/*v*) Triton X-100 was required to solubilize the substrates. This concentration of non-ionic detergent along with increased substrate concentrations may have facilitated the formation of mixed micelles, which increased the interfacial activation effect. The differences between our data and other recent determinations of kinetic parameters could be due to variations in the assay conditions. However, given the similar concentration of substrate and Triton X-100 used—both well above their critical micelle concentration (CMC)—the issue may lie more in the criteria applied to assess a model that agrees with the experimental data.

There are kinetic models that aim to better fit the characteristics of lipases in interfaces compared to the Michaelis–Menten steady-state model. However, these models require a comprehensive characterization of the micelles formed in the assay, such as the total interface area of micelles or the substrate concentration in the interface, and are usually non-steady-state models [60,61,62]. Marangoni (1994) proposed a simplified model for enzyme kinetics of lipolysis, whose derived equation resembled the allosteric sigmoidal model (Hill equation) [59]. This model takes into account that the rate-limiting step of the reaction is the interfacial binding step in which the enzyme binds to a group of substrate molecules, leading to a conformational change. This binding is in a “hopping mode,” meaning that the enzyme can leave the interface with a specific dissociation constant, which depends not only on the substrate structure but also on the “interfacial quality” of the surface where the substrate is arranged. The binding is a cooperative process involving conformational changes in the enzyme, structural changes in the substrate interface, and penetration of the lipase into that substrate interface. Similar to steady-state models, this model is applicable at low enzyme–interface ratios and early stages in the reaction (initial velocity). *K*_1/2_ is the dissociation constant for the lipase interface, and the Hill coefficient (n) is the number of lipid molecules associated with the catalytic process per catalytic cycle, or more realistically, it can be interpreted as an index of the cooperativity of the entire process.

Although some recent publications have applied this model for the characterization of lipases, it is still not widely used [63,64]. However, we consider that it is a better model for fitting enzyme saturation curves for lipid substrates, requiring attention to consider that the parameters obtained are more related to the nature of the interface than to the intrinsic nature of the substrate. In our case, we can preliminarily consider that Lip7 has a higher affinity for interfaces composed of medium-chain substrates, but in order to confirm this, more experiments under varying conditions are needed (e.g., with different emulsifiers and detergents). In addition, it could be interesting to evaluate whether this result can be extrapolated to reactions more commonly used in the industry, such as transesterification for biodiesel production.

Considering all of the above, lipase activity is highly dependent on the assay conditions used, and in order to compare results fairly, it is best to empirically determine the kinetic parameters of different lipases using the same enzyme assay. Additionally, as previously mentioned, the majority of the reported kinetic data for lipases are directly fitted to the Michaelis–Menten model [13,52,53,54,55]. However, a comparison can still be made regarding the maximum velocity, as reported maximum velocity ranges for lipases using pNPL (C12) range from 200 to 5000 U mg^−1^ [49,52,65]. Lip7 exhibits a maximum velocity of 491 U mg^−1^ at 25 °C, pH 8.0, and a maximum velocity of 3350 U mg^−1^ at 50 °C, pH 11.0, which are within the reported range for other lipases.

Finally, Lip7 was shown to be stable in the presence of organic solvents, as it maintained at least 30% of its activity, making it an interesting candidate for use in biocatalytic processes requiring high concentrations of organic solvents. Moreover, in some cases, Lip7 even undergoes enzymatic activation, reaching up to 2.5-fold and 1.35-fold when incubated in 50% *v*/*v* ethanol and 70% *v*/*v* isopropanol, respectively. Similar results have been reported for other lipases, showing increases in activity ranging from 20% to 200% [66,67,68]. One explanation for this effect with solvents is that incubation increases the structural flexibility of the lid over the active site, facilitating substrate entry [68]. The increased flexibility of the lid could also explain the enzymatic activation observed when Lip7 was incubated at 40 °C, pH 8.0 and pH 11.0 (Figure 3). However, this same increased flexibility may lead to enzyme denaturation, explaining the differences in each condition. Therefore, enzyme activation may represent an equilibrium between increased flexibility and total denaturation, depending on the solvent structure and concentration used.

Similarly, when immobilizing strategies have been used for lipases, as seen in *Thermomyces lanuginosus* and *Rhizomucor miehei* lipases, changes in the immobilization conditions can modify properties such as substrate specificity, activity, and stability [69,70]. This suggests that the medium conditions could affect the enzyme structure, and then immobilization would maintain these conformations. Thus, Lip7 could be an interesting candidate for further studies on the enzymatic immobilization process, with the idea of immobilizing its activated conformation after incubation in solvents.

## 4. Materials and Methods

### 4.1. Recombinant Expression of Lip7 Lipase

The putative lipase encoding gene (*lip7*) from *Geobacillus* sp. ID17 was synthesized and subcloned into pET-28a plasmid (GenScript, Piscataway, NJ, USA) without its predicted signal peptide sequence. This plasmid contains the T7 promotor, and a kanamycin resistance gene for selection.

Competent cells of *Escherichia coli* strains BL21 (DE3) (Thermo Fisher Scientific, Waltham, MA, USA) and OverExpress™ C41 (DE3) (Sigma Aldrich, St. Louis, MO, USA) were chemically transformed with pET-28a-*lip7*. Transformants were grown aerobically in 400 mL of autoinduction TBA culture medium supplemented with 50 μg mL^−1^ kanamycin (USBiological, Salem, MA, USA) at 30 °C, with shaking at 180 rpm for 30 h [40,71]. Then, the cells were harvested by centrifugation (Himac CP80WX, Hitachi Koki, Tokyo, Japan) at 12,400× *g* for 15 min at 4 °C and resuspended in 50 mM Tris-HCl buffer, pH 8.0 supplemented with 10% glycerol. Cell disruption was performed by sonication (Branson 450 Digital Sonifier, Marshall Scientific, Hampton, NH, USA), followed by centrifugation of the cell lysate at 22,000× *g* for 20 min at 4 °C to obtain the soluble crude extract.

Lipase overexpression was evaluated by 12% sodium dodecyl sulfate–polyacrylamide gel electrophoresis (SDS-PAGE) and visualized by staining with Coomassie brilliant blue R-250 [72]. Protein concentration was determined using Bio-Rad protein assay (Bio-Rad, Inc., Hercules, CA, USA) with bovine serum albumin (BSA) protein standard solution of 200 mg mL^−1^ (Sigma Aldrich, St. Louis, MO, USA) as the reference. Measurements were performed on a BioTek EPOCH2 microplate spectrophotometer (BioTek, Winooski, VT, USA) at 595 nm.

### 4.2. Lipase Activity Assay

Lipase activity was routinely assayed spectrophotometrically following the appearance of p-nitrophenol (pNP) released as a result of the hydrolysis of the chromogenic substrate p-nitrophenyl laurate (pNPL, C12) from Sigma-Aldrich (Sigma Aldrich, St. Louis, MO, USA).

The enzyme assays were performed following previously described protocols with some modifications [73,74]. Miniaturized assays were conducted in a volume of 200 µL containing 175 µL of buffer with 2% (*v*/*v*) Triton X-100, 3.0 mM of pNPL substrate dissolved in acetonitrile, and 5 μL of lipase sample on 96-well flat-bottom microplates (Thermo Fisher Scientific, Waltham, MA, USA). The reaction was initiated by the addition of substrate after 5 min preincubation of the reaction mixture at the desired temperature, and all assays were conducted in triplicate.

The reaction was monitored by measuring the change in absorbance at 405 nm over time using a BioTek EPOCH2 microplate spectrophotometer (BioTek, Winooski, VT, USA). Additionally, the change in absorbance was also measured at 347 nm, as the absorbance of pNP at that wavelength is independent of the pH [75]. The concentration of pNP formed during the assay was determined using calibration curves with a 10 mM pNP standard (Sigma-Aldrich, St. Louis, MO, USA). Increasing concentrations of 5, 25, 50, 100 and 200 μM were used, and the absorbance was measured at both wavelengths (405 and 375 nm).

One unit (U) of lipase activity is defined as the release of 1 μmol of pNP per min under the assay conditions.

### 4.3. Protein Purification

The recombinant Lip7 lipase was purified by a first step of heat-denaturation treatment, which precipitates thermosensitive native proteins from *E. coli*, allowing easy partial purification of Lip7. The effect of salt addition on increasing Lip7 stability was evaluated by incubating the solution in 0.5 mM CaCl_2_, ZnCl_2_ or a combination of both salts for 15 min at 25 °C prior to heat-denaturation treatment at 60 °C for 30 min (Figure S2). The enriched soluble crude extract was then obtained by centrifugation at 22,100× *g* for 20 min at 4 °C, and concentrated using an Amicon ultrafiltration stirring cell (Merck Millipore, Burlington, MA, USA) with a 10 kDa ultrafiltration membrane disk (Ultracel, Merck Millipore, Burlington, MA, USA).

Subsequent purification steps were carried out at room temperature on a fast-performance liquid chromatography (FPLC) system (Pharmacia Biotech, Stockholm, Sweden). For cation exchange, the soluble heat-denatured crude extract was diluted 10-fold in loading buffer (50 mM MES, pH 6.0) and loaded onto a pre-equilibrated 1 mL Hitrap SP-Sepharose Fast Flow column (Cytiva, Marlborough, MA, USA) at a flow rate of 1 mL min^−1^. The column was washed with 5 column volumes (CV) of the same buffer at 1 mL min^−1^, and the enzyme was eluted by a linear NaCl gradient (0–0.5 M) at a flow rate of 1 mL min^−1^. Fractions containing lipase activity were pooled and concentrated using an Amicon Ultra 15 (30 kDa MWCO) centrifugal filter device (Merck Millipore, Burlington, MA, USA). The purified Lip7 fraction was stored in buffer 50 mM Tris-HCl pH 8.0 at −20 °C with the addition of 10% glycerol.

Size-exclusion chromatography was performed as a final purification step and to determine the molecular mass and oligomeric state of the recombinant lipase. The cation exchange-purified Lip7 was loaded onto a 102 mL Superdex 200 Tricorn 10/600 XK-16/70 column (General Electric™ Healthcare, Boston, MA, USA) pre-equilibrated with 10 CV of 50 mM Tris-HCl buffer pH 8.0 containing 0.2 M NaCl at 1 mL min^−1^ flow rate, and was eluted in the same conditions. The column was calibrated using carbonic anhydrase (29 kDa), ovalbumin (43 kDa), bovine serum albumin (BSA) (66 kDa), conalbumin (75 kDa), and blue dextran (2000 kDa) (gel filtration calibration kit LMW, General Electric™ Healthcare, Boston, MA, USA; Sigma Aldrich, St. Louis, MO, USA) as standards.

The apparent molecular mass of the protein was also estimated by SDS-PAGE by determining the relative migration distance of the molecular weight marker (Bio-Rad Precision Plus Protein™ Kaleidoscope™ pre-stained protein standard (Bio-Rad, Inc., Hercules, CA, USA)) and the recombinant lipase. The processing of SDS-PAGE images was performed using ImageJ software version 1.54i (National Institutes of Health, Bethesda, MD, USA, Public Domain) [28].

### 4.4. Biochemical Characterization of the Purified Recombinant Enzyme

#### 4.4.1. Effect of pH

The influence of pH on the catalytic activity of the recombinant lipase was determined with pNPL substrate. Miniaturized assays were conducted as described in Section 4.2, at 50°C using Britton–Robinson universal buffer (40 mM boric acid, 40 mM phosphoric acid, and 40 mM acetic acid) [76] with 2% (*v*/*v*) Triton X-100 adjusted at different pH in a range of 4.0–13.0 with NaOH, 3 mM of pNPL substrate, and 0.25 nM (0.011 μg mL^−1^) of purified Lip7.

#### 4.4.2. Effect of Temperature

The temperature dependence of the recombinant lipase was determined with pNPL substrate. Miniaturized assays were conducted as described in Section 4.2, over the temperature range of 30–65 °C at different pH (8.0–12.0) using Britton–Robinson universal buffer with 2% (*v*/*v*) Triton X-100, 3 mM of pNPL substrate, and 0.25 nM (0.011 μg mL^−1^) of purified Lip7.

Thermal stability of the recombinant lipase was determined by incubating 1 μM of Lip7 at 40 °C, 50 °C, 60 °C, and 70 °C for 0, 0.25, 0.5, 1, 2, and 4 h in 50 mM Tris-HCl buffer, pH 8.0, and Britton–Robinson universal buffer, pH 11.0. After each incubation period, the samples were promptly cooled on ice for 5 min and assayed as described in Section 4.2, at 50 °C and pH 11.0 using Britton–Robinson buffer with 2% (*v*/*v*) Triton X-100, 3 mM of pNPL substrate, and 0.25 nM (0.011 μg mL^−1^) of purified Lip7. The residual activity was obtained by comparison with the non-heated control, which represents a relative activity of 100%.

#### 4.4.3. Stability against Organic Solvents

The influence of different organic solvents—methanol, ethanol, isopropanol, acetonitrile, and acetone (all from JT Baker^®^, Philadelphia, PA, USA)—on the enzyme activity was determined by incubating 1 μM of Lip7 in different solvent–water concentrations (%*v*/*v*): 0%, 10%, 20%, 40%, 50%, 70%, and 90%, for 30 min at 25 °C. After each incubation period, the samples were promptly cooled on ice for 5 min and assayed as described in Section 4.2, at 50 °C, pH 11.0 using Britton–Robinson buffer with 2% (*v*/*v*) Triton X-100, 3 mM of pNPL substrate, and 0.25 nM (0.011 μg mL^−1^) of purified Lip7. The residual activity was obtained by comparison with the non-incubated control, which represents a relative activity of 100%.

#### 4.4.4. Substrate Specificity

Catalytic activity of Lip7 was also assayed with substrates of different chain-length—p-nitrophenyl acetate (pNPA, C2), p-nitrophenyl octanoate (pNPO, C8), p-nitrophenyl decanoate (pNPD, C10), pNPL (C12), p-nitrophenyl myristate (pNPM, C14), and p-nitrophenyl palmitate (pNPP, C16)—all obtained from Sigma-Aldrich (Sigma Aldrich, St. Louis, MI, USA).

Miniaturized assays were conducted as described in Section 4.2, at 25 °C, in 50 mM potassium phosphate buffer pH 8.0 with 2% (*v*/*v*) Triton X-100, as short-chain substrates hydrolyze spontaneously at higher temperatures and pH.

Initial velocities were fitted to both the Michaelis–Menten and allosteric sigmoidal models using GraphPad Prism software (version 8.0.2, GraphPad Software, Boston, MA, USA, 2019) to determine the kinetic parameters for each substrate.

### 4.5. Statistical Analysis

The statistical analysis was performed using GraphPad Prism software (version 8.0.2, GraphPad Software, Boston, MA, USA, 2019). The results are expressed as the mean of triplicates ± standard deviation (SD).

## Figures and Tables

**Table 1:** Purification of recombinant Lip7 from 400 mL culture of *E. coli* C41 (DE3) grown in TBA medium. Enzyme activity was measured at 50 °C, pH 11.0 using p-nitrophenyl laurate (pNPL) substrate.

Purification Step	Total Protein (mg)	Total Activity (U)	Specific Activity (U mg^−1^)	Yield (%)	Purification (Fold)
Soluble crude extract	280 ± 20	220,000 ± 33,000	790 ± 120	100	1
Heat treatment (60 °C × 30 min)	62 ± 4	110,000 ± 15,000	1800 ± 250	50 ± 7	2.6 ± 0.3
Cation exchange chromatography	18 ± 1	46,000 ± 5000	2600 ± 280	21 ± 2	3.3 ± 0.4
Size-exclusion chromatography	15 ± 2	50,000 ± 1000	3400 ± 80	23.0 ± 0.6	4.3 ± 0.1

**Table 2:** Kinetic parameters for different chain-length substrates obtained from allosteric sigmoidal model. Enzyme activity was measured at 25 °C, pH 8.0.

Substrate	*V*_max_ (U mg^−1^)	Hill Coefficient (n)	*K*_1/2_ (mM)	*k*_cat_ (s^−1^)	*k*_cat_/*K*_1/2_ (s^−1^mM^−1^)	R^2^ Correlation
pNPA (2C)	772 ± 54	1.9 ± 0.5	3.4 ± 0.7	556 ± 39	163	0.90
pNPO (8C)	229 ± 21	2.4 ± 0.8	1.6 ± 0.3	165 ± 15	103	0.82
pNPD (10C)	466 ± 39	3.1 ± 1.4	2.2 ± 0.3	336 ± 28	153	0.85
pNPL (12C)	491 ± 93	1.8 ± 0.4	1.7 ± 0.4	354 ± 67	208	0.98
pNPM (14C)	201 ± 24	3.2 ± 0.6	1.4 ± 0.1	145 ± 17	104	0.97
pNPP (16C)	(100) *	---	---	---	---	---

* For a substrate concentration of 2.0 mM pNPP, the velocity obtained was 100 U mg^−1^ before its low solubility impeded measurement at higher concentrations.

## Data Availability

The data presented in this study are available on request from the corresponding author. The data are not publicly available due to privacy reasons.

## Supplementary Materials

The following supporting information can be downloaded at https://www.mdpi.com/article/10.3390/ijms25147928/s1.
