# Supplementary material for: Recombinant Expression and Characterization of a Novel Thermo-Alkaline Lipase with Increased Solvent Stability from the Antarctic Thermophilic Bacterium Geobacillus sp. ID17
Source: Int J Mol Sci. 2024 Jul 19;25(14):7928. doi: 10.3390/ijms25147928 (PMC11277018; doi:10.3390/ijms25147928)
Supplement: Supplementary file 1 [file ijms-25-07928-s001.zip › ijms-3062736-supplementary.pdf]

International Journal of
Molecular Sciences

MDPI

Supplementary Material

Recombinant Expression and Characterization of a Novel Thermo-Alkaline Lipase with Increased Solvent Stability from the Antarctic Thermophilic Bacterium *Geobacillus* sp. ID17

Diego Salas-Bruggink[1], Hardy Guzmán[2], Giannina Espina[1]*, and Jenny M. Blamey[1,2,]*

[1] Fundación Biociencia, José Domingo Cañas 2280, Santiago 7750132, Chile; dsalas@bioscience.cl
[2] Facultad de Química y Biología, Departamento de Biología, Universidad de Santiago de Chile, Alameda 3363, Santiago 9170022, Chile; hardy.guzman@usach.cl
* Correspondence: jblamey@bioscience.cl, gespina@biosciencia.cl

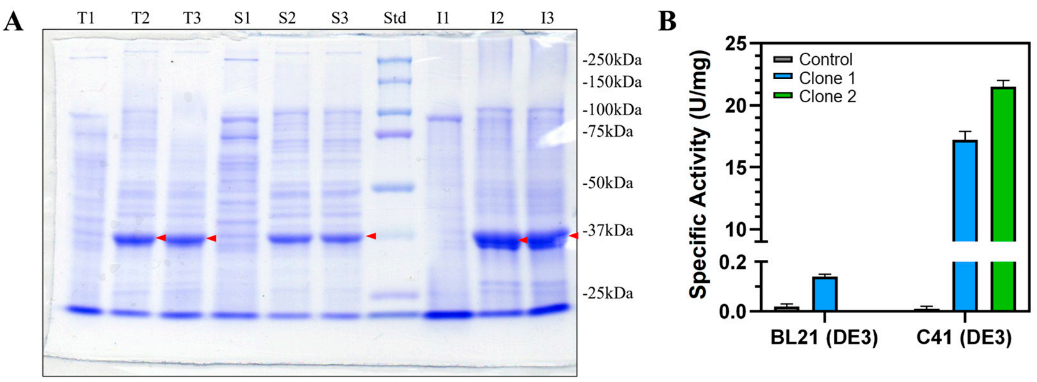

Figure S1. Evaluation of *E. coli* BL21 (DE3) and two clones of *E. coli* C41 (DE3) for the recombinant expression of Lip7 in functional soluble form. (A) Electrophoretic analysis. Lane T1: *E. coli* BL21 (DE3) lysate (total fraction). Lane T2: *E. coli* C41 (DE3) (clone 1) lysate. Lane T3: *E. coli* C41 (DE3) (clone 2) lysate. Lane S1: *E. coli* BL21 (DE3) cell-free soluble crude extract. Lane S2: *E. coli* C41 (DE3) (clone 1) cell-free soluble crude extract. Lane S3: *E. coli* C41 (DE3) (clone 2) cell-free soluble crude extract. Lane Std: molecular weight marker (Bio-Rad Precision Plus Protein™ Kaleidoscope™ pre-stained protein standard (Bio-Rad, Inc., Hercules, CA, USA)). Lane I1: *E. coli* BL21 (DE3) inclusion bodies solubilized with 8 M urea (insoluble fraction). Lane I2: *E. coli* C41 (DE3) (clone 1) inclusion bodies solubilized with 8 M urea. Lane I3: *E. coli* C41 (DE3) (clone 2) inclusion bodies solubilized with 8 M urea. The protein concentration loaded in each well is 15 µg and the band corresponding to Lip7 is highlighted by red arrows. (B) Enzyme activity of Lip7 in cell-free soluble crude extracts (S1, S2, S3). The assays were performed at 20°C, pH 8.0, using 1.5 mM pNPL as substrate. A non-transformant control was also assayed for each strain.

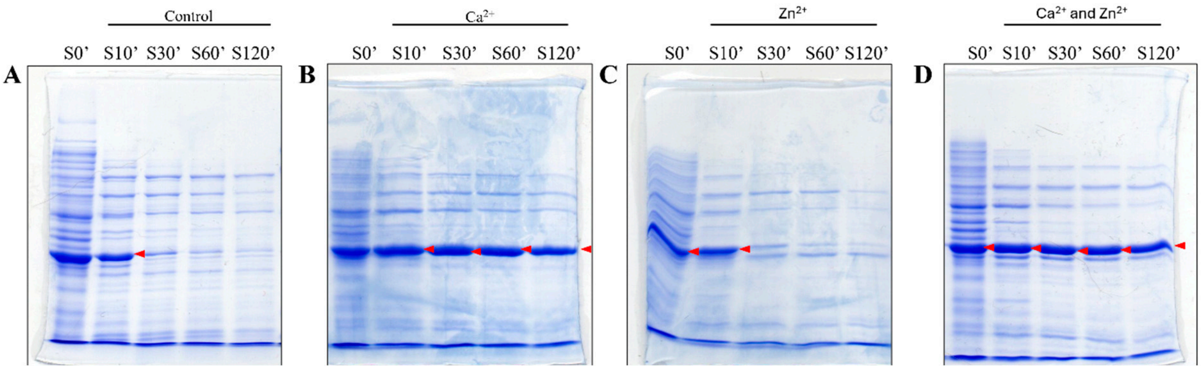

Figure S2. Electrophoretic analysis of the effect of Ca^{2+} and Zn^{2+} in the thermal stability of Lip7 during heat denaturation treatment. The soluble crude extract was incubated at 60°C for 0, 10, 30, 60, and 120 min (lanes S0', S10', S30', S60', and S120') after preincubation with calcium and zinc salts for 15 min at 25°C. (A) no preincubation control; (B) 0.5 mM $CaCl_2$; (C) 0.5 mM $ZnCl_2$; (D) 0.5 mM $CaCl_2$ and 0.5 mM $ZnCl_2$. The protein concentration loaded in each well is 15 µg and the band corresponding to Lip7 is highlighted by red arrows.

Table S1. Kinetic parameters for different chain-length substrates obtained from Michaelis-Menten model. Enzyme activity was measured at 25°C, pH 8.0.

**Substrate**	V_{max} **(U mg^{-1})**	K_m **(mM)**	k_{cat} **(s^{-1})**	$k_{cat}/K_{1/2}$ **(s^{-1}mM^{-1})**	**R^2 correlation**
pNPA (2C)	961 ± 94	5.3 ± 0.2	692 ± 68	131	0.87
pNPO (8C)	310 ± 51	2.7 ± 1.1	223 ± 37	83	0.77
pNPD (10C)	707 ± 145	4.5 ± 2.0	509 ± 104	113	0.80
pNPL (12C)	2036 ± 1228	13.3 ± 9.5	1466 ± 884	110	0.96
pNPM (14C) *	----	----	----	----	----
pNPP (16C) *	----	----	----	----	----

* The data obtained for pNPM and pNPP could not be fitted to the Michaelis-Menten model.

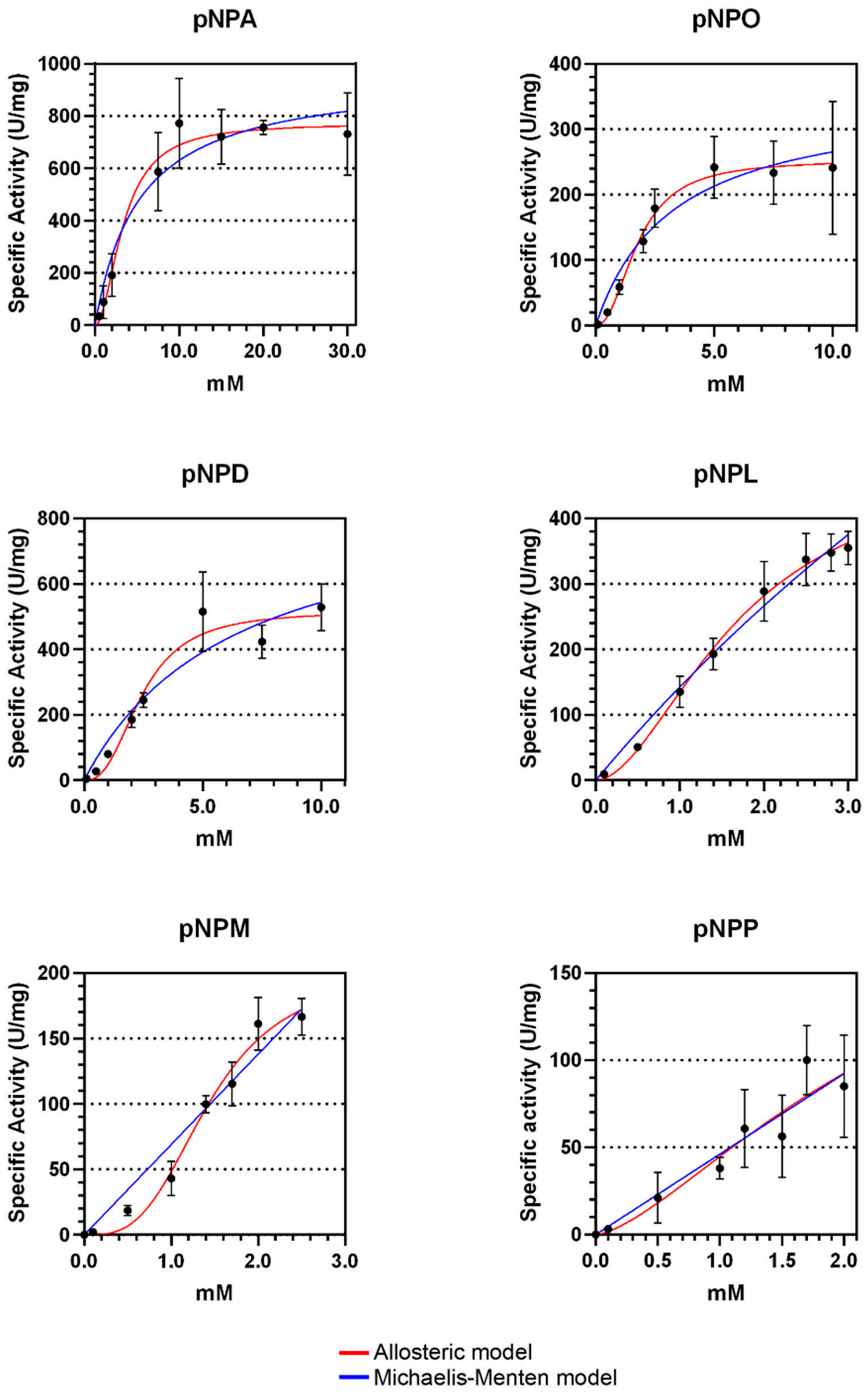

Figure S3. Kinetic curves of Lip7 with different chain-length substrates: p-nitrophenyl acetate (pNPA, C2), p-nitrophenyl octanoate (pNPO, C8), p-nitrophenyl decanoate (pNPD, C10), p-nitrophenyl laurate (pNPL, C12), p-nitrophenyl myristate (pNPM, C14), and p-nitrophenyl palmitate (pNPP, C16). Enzyme activity was measured at 25°C, pH 8.0. Initial velocities were fitted to both the Michaelis-Menten and allosteric sigmoidal models using GraphPad Prism software (version 8.0.2, GraphPad Software, Boston, MA, USA, 2019).
